# Enhancing the heat tolerance of reef-building corals to future warming

**DOI:** 10.1126/sciadv.abg6070

**Published:** 2021-08-20

**Authors:** Emily J. Howells, David Abrego, Yi Jin Liew, John A. Burt, Eli Meyer, Manuel Aranda

**Affiliations:** 1 Water Research Center and Center for Genomics and Systems Biology, New York University Abu Dhabi, Abu Dhabi, United Arab Emirates.; 2 Centre for Sustainable Ecosystem Solutions, School of Earth, Atmospheric and Life Sciences, University of Wollongong, Wollongong, New South Wales, Australia.; 3 Department of Natural Science and Public Health, Zayed University, Abu Dhabi, United Arab Emirates.; 4 National Marine Science Centre, Faculty of Science and Engineering, Southern Cross University, Coffs Harbour, New South Wales, Australia.; 5 CSIRO Health and Biosecurity, North Ryde, New South Wales, Australia.; 6 Department of Integrative Biology, Oregon State University, Corvallis, OR, USA.; 7 Division of Biological and Environmental Sciences and Engineering, King Abdullah University of Science and Technology, Thuwal, Kingdom of Saudi Arabia.

## Abstract

Reef-building corals thriving in extreme thermal environments may provide genetic variation that can assist the evolution of populations to rapid climate warming. However, the feasibility and scale of genetic improvements remain untested despite ongoing population declines from recurrent thermal stress events. Here, we show that corals from the hottest reefs in the world transfer sufficient heat tolerance to a naïve population sufficient to withstand end-of-century warming projections. Heat survival increased up to 84% when naïve mothers were selectively bred with fathers from the hottest reefs because of strong heritable genetic effects. We identified genomic loci associated with tolerance variation that were enriched for heat shock proteins, oxidative stress, and immune functions. Unexpectedly, several coral families exhibited survival rates and genomic associations deviating from origin predictions, including a few naïve purebreds with exceptionally high heat tolerance. Our findings highlight previously uncharacterized enhanced and intrinsic potential of coral populations to adapt to climate warming.

## Teaser

Selective breeding increases the resilience of reef-building corals to climate warming.

## INTRODUCTION

**Fig. 1:**
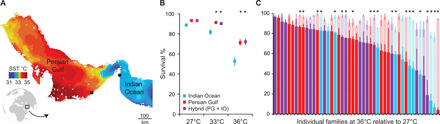
Location of source coral populations and heat tolerance of their larval offspring. (**A**) Summer sea surface temperature (SST) in the Persian Gulf (PG) and Oman Sea in the Indian Ocean (IO) (mean of 5 km nighttime SST for August 2015, National Oceanic and Atmospheric Administration; https://coralreefwatch.noaa.gov/product/5km/index.php). Markers (black circles) indicate the location of collection sites of *P. daedalea* coral fragments at Saadiyat Island (PG) and Al Aqah Island (IO). (**B**) Effect of parental origin on the survival (means ± SE) of *P. daedalea* after 60 hours of exposure to ambient (27°C) and elevated (33° and 36°C) temperatures. (**C**) Variation in survival rates (means ± SE) among individual *P. daedalea* families after 60 hours of exposure to 36°C (foreground) relative to 27°C (background). PG families are represented in red, IO families in blue, and regional hybrids (PG × IO) in purple. Asterisks indicate significant effects (*P <* 0.05) of parental origin and family on survival at elevated temperature. For analyses, survival rates were standardized to ambient values for each family to account for mortality unrelated to heat exposure and unequal variances. Parental origin (IO, PG, and PG × IO) effects were analyzed with a linear model (elevated temperature*origin) with Tukey post hoc comparisons. Individual family effects at 36°C were analyzed using one-tailed (<1) *t* tests with adjusted *P* values (false discovery rate correction).

Catastrophic consequences for global coral reef ecosystems are predicted under even the most optimistic emission scenarios, with 70 to 90% of reef-building corals expected to die under global warming of 1.5°C (*1*). Reef-building corals are especially vulnerable to population declines and extinctions under anthropogenic warming (*2*) as they live very close to their upper thermal limits (*3*). However, thermal heterogeneity across species ranges drives local adaptive variation in the absolute temperatures that cause thermal stress and mortality (*4*), and this variation potentially provides genetic material to selectively breed for enhanced thermal tolerance. In the hottest region for reef-building corals, in the young Persian (Arabian) Gulf sea [≤6 thousand years (ka)] (*5*), coral populations have already adapted to thermal maximums not expected on most tropical reefs until the end of this century (*6*, *7*). Sea temperatures typically remain above 34°C for approximately eight consecutive weeks in summer and reach highs of 35° to 36°C (Fig. 1A) (*6*), exceeding the summer maxima and thermal limits of almost all coral populations elsewhere in the world by more than 2°C (*7*). Selection for tolerant genotypes in the Persian Gulf likely occurs in the presence of migration between neighboring Indian Ocean coral populations, which typically experience cooler 30° to 31°C summers and have been naïve to temperatures exceeding 34°C in recent history and likely also the past 25 ka (*6*, *8*).

## RESULTS AND DISCUSSION

Here, we evaluated the scope of heat-adapted Persian Gulf corals to act as a genetic resource to enhance the resilience of coral populations to climate warming. The widespread brain coral, *Platygyra daedalea*, was bred from parents either experienced (Persian Gulf) or naïve (Indian Ocean) to end-of-century temperatures (*6*). In total, 50 families were bred, comprising 30 regional purebreds and 20 intraspecific hybrids between Persian Gulf fathers and Indian Ocean mothers (fig. S1). Family variation in heat tolerance was measured by tracking the survival of >12,000 individual larvae at ambient temperature (27°C) and two elevated temperatures (33° and 36°C) representing the annual maximum in each region (table S1). As expected, coral survival was negatively affected by exposure to elevated temperatures, with the greatest mortality occurring in Indian Ocean purebred families (Fig. 1B). While there was no regional parental effect on survival at 27°C, the mortality of Indian Ocean purebreds at 33° and 36°C was higher than of Persian Gulf purebreds by 52 and 40%, respectively (*P <* 10^−2^; table S3A). Yet, when Indian Ocean mothers were bred with Persian Gulf fathers, heat survival was enhanced by 37% and was (on average) equivalent to that of Persian Gulf purebreds (Fig. 1B). This represents a tolerance enhancement to future temperature maxima in the Indian Ocean projected for midcentury under worst-case emission scenarios [+2.0°C, Representation Concentration Pathway (RCP) 8.5] and end-of-century under intermediate scenarios (+2.2°C, RCP 6.0) (table S1) (*9*). Regional patterns of heat tolerance were accompanied by sizeable phenotypic variation among individual families (Fig. 1C). After 2.5 days at 36°C, most Indian Ocean purebred families had survival rates of <60% (53% on average). However, a few families had unexpectedly high survival rates of >80% despite both parents being naïve to extreme temperatures. The heat tolerance of these exceptional Indian Ocean families was greater than the mean response of Persian Gulf purebreds (71%) and regional hybrids (72%). Conversely, a few families with one or both parents from the extreme Persian Gulf exhibited unexpected heat sensitivity including the family with the lowest recorded survival (4%). The combined effect of both parents accounted for 75% of the observed variation in heat tolerance (η_p_^2^ family = 0.75, *P <* 10^−14^; table S3B).

**Fig. 2:**
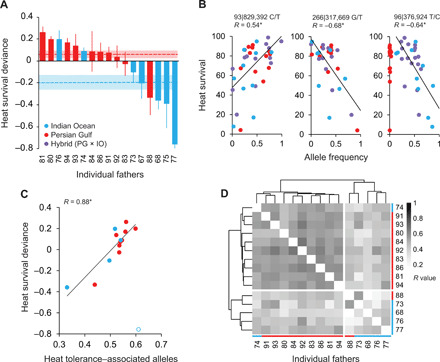
Paternal contributions to the heat tolerance of coral offspring. (**A**) Relative gains and losses in heat survival (means ± SE) observed in larval families of *P. daedalea* bred with fathers from populations experienced (Persian Gulf, red) or naïve (Indian Ocean, blue) to future warming. Values are survival deviance at 36°C expressed relative to maternal means. Individual and regional values are columns and dashed lines, respectively. (**B**) Representative correlations between SNP minor allele frequencies (MAFs) and heat survival (Bonferroni-corrected *P <* 0.05, indicated by asterisks). Either the strongest associations were consistent across families or the allele associated with enhanced heat survival was fixed (or near fixed) in Persian Gulf families. In total, 4341 SNPs were significantly associated with heat survival. (**C**) The capacity of individual fathers to produce heat-tolerant offspring was underpinned by their genotypes. SNPs associated with heat tolerance (*n* = 4341) were identified from families [see (B)], and the average proportion of beneficial alleles was calculated for each paternal genotype (see Materials and Methods). The proportion of alleles was positively associated with survival deviance (*P <* 0.05, indicated by asterisk), and the correlation coefficients (*R*) for all fathers, Indian Ocean fathers, and Persian Gulf fathers are 0.88, 0.94, and 0.97, respectively. Correlations exclude one outlying Indian Ocean father (77, open circle), which was only represented by a single cross. (**D**) Clustering of the pairwise correlation (grayscale values) of paternal genotypes at SNPs strongly associated with heat survival (*R* ≤ −0.5 and ≥ 0.5, *n =* 542) highlights deviations from regional population differentiation (fig. S2) corresponding to paternal performance.

Gains in the heat tolerance of regional hybrids were expected to have a genetic basis as Persian Gulf genes were introduced paternally to eggs of the same mothers used for purebred Indian Ocean crosses. This treatment controlled for any confounding effect of nongenetic maternal factors that can influence offspring phenotypes, although these had a minor effect in our study (η_p_^2^ mother = 0.21, *P <* 10^−4^; table S3C). In contrast, the identity of individual fathers accounted for 52% of the variation in heat tolerance (η_p_^2^ father = 0.52, *P <* 10^−9^; table S3C). Estimates of survival heritability (*h*^2^) therefore considered the increased phenotypic similarity of offspring from the same mother and ranged from 0.46 to 0.65 at 36°C but did not differ from zero at 27°C (table S4). In line with regional expectations, most Persian Gulf fathers (8 of 10) produced offspring with tolerance gains and most Indian Ocean fathers (5 of 7) produced offspring with tolerance losses (Fig. 2A). However, one Persian Gulf father produced offspring with tolerance below the mean Indian Ocean phenotype, and two Indian Ocean fathers produced offspring with tolerance equivalent to or exceeding the mean Persian Gulf phenotype. Together, these findings demonstrate strong regional and individual genetic differences in heat tolerance that are readily transferrable to offspring via selective breeding.

To understand the genomic basis for the observed phenotypic variation, we identified single-nucleotide polymorphisms (SNPs) that were associated with heat survival and responded to heat selection. Restriction site–associated DNA sequencing (2b-RAD) (*10*) was performed on coral larvae that survived the temperature experiment [*n* *=* 87 family × temperature samples, after quality control (QC)] and their parents (*n =* 15, after QC). SNPs were identified by the alignment of sequencing reads to the annotated *P. daedalea* genome (*11*). Allele frequencies in coral larval families at ambient temperature were predictive of survival rates at elevated temperature. Of 38,519 SNPs tested (≥50× coverage) with parental origin as a random factor, 542 were strongly predictive (1.4%; *R* ≤ −0.5 and ≥ 0.5) and a further 13,896 were moderately to weakly predictive (36%). The strongest associations either showed consistent trends across families regardless of parental origin or fixation (or near fixation) of alleles associated with enhanced heat survival in Persian Gulf purebreds (Fig. 2B). In parental samples, genotypes across these SNPs were also associated with the ability to sire heat-tolerant offspring (Fig. 2C), where the average proportion of beneficial alleles (0.33 to 0.61) explained the majority of performance variation among individual fathers (*R* = 0.88, *P <* 10^−4^). The Persian Gulf father that unexpectedly produced heat-sensitive offspring (PG88) had a low proportion of beneficial alleles (0.44) and was genetically similar to one of the heat-sensitive Indian Ocean fathers across SNPs strongly associated with heat survival (Fig. 2D). In addition, the Indian Ocean fathers that unexpectedly produced heat-tolerant offspring (IO74 and IO76) had a relatively high proportion of beneficial alleles (0.52 to 0.54) and were more genetically similar to one (IO76) or several (IO74) heat-tolerant Persian Gulf fathers than the remaining Indian Ocean fathers. This demonstrates that beneficial alleles prevalent in the Persian Gulf are also present within the Indian Ocean population. If the young Persian Gulf population of *P. daedalea* evolved its exceptional heat tolerance from standing genomic variation, then beneficial ancestral variants may be present at cryptic levels within older and cooler Indian Ocean populations, as has been shown for the dinoflagellate symbionts of these corals (*12*). Alternatively, beneficial ancestral or derived variants in Persian Gulf populations could be transported to the Indian Ocean via the dispersal of larvae in wind-driven surface currents and prevailing bottom water currents exiting the Gulf (*13*).

**Fig. 3:**
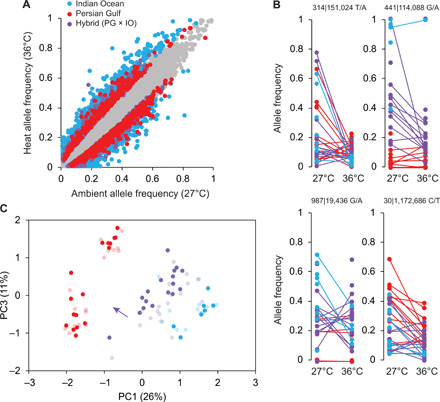
Genomic signatures of heat selection in families of coral larvae bred from parents naïve or experienced to future warming. (**A**) Differences in the MAF of SNPs (circles) in Persian Gulf (PG, red), Indian Ocean (IO, blue), and regional hybrid (PG × IO, purple) families of *P. daedalea*, shown by deviation from the diagonal. Colored data points show SNPs with significant differences in between temperatures (Bonferroni-corrected *P <* 0.05) with the strongest selection response in IO families (greatest deviation and 9.3% of SNPs significant, *n =* 16,792), followed by PG × IO hybrids (5.0%, *n =* 20,307) and PG families (3.7%, *n =* 30,095). (**B**) MAFs in individual families (lines) at representative SNPs with significant temperature effects. Responses included selection against alleles common to families regardless of their parental background and those that were mediated by parental origin effects. (**C**) Principal components (PCs) of MAFs at SNPs that had significant allele frequency differences at 36°C versus 27°C in selectively bred hybrid coral families [PG × IO see (A)]. SNPs were filtered by *P* value (Bonferroni-corrected *P <* 0.001) and a missing data threshold of less than 10% across all families (*n =* 147). Remaining missing values were replaced by regional (IO, PG, or hybrid) means. The general shift of hybrid families at 36°C (dark purple) versus 27°C (light purple) away from Indian Ocean families (blue) and toward Persian Gulf families (red) demonstrates that beneficial alleles were predominantly inherited from Persian Gulf fathers rather than Indian Ocean mothers.

Selection for more tolerant genotypes was indicated in our experiment by significant shifts in allele frequencies in *P. daedalea* families at 36°C versus 27°C. Predictably, the highest proportion of temperature-differentiated SNPs (9.3% of 19,792 SNPs) and the greatest extent of allele frequency shifts (±0.16 on average) occurred in Indian Ocean purebreds (Fig. 3A), which experienced the highest mortality (Fig. 1B). Selection effects were comparatively weaker in Persian Gulf purebreds (3.7% of 30,095 SNPs, by ±0.10 on average). Despite experiencing the same level of mortality, regional hybrids showed slightly higher selection response than the Persian Gulf purebreds (5.0% of 19,792 SNPs, by ±0.11 on average). Responses included selection for and against alleles common to families regardless of their parental background and those that were mediated by parental origin effects (Fig. 3B). Origin effects were predominantly characterized by heat selection for alleles that were more prevalent, and in some instances fixed, in Persian Gulf purebreds. For example, heat-selected alleles were 89% more likely to be fixed (frequency < 0.01) in Persian Gulf families than in Indian Ocean families. The predominant Persian Gulf origin of selected alleles is illustrated in hybrid families, which became genetically more similar to Persian Gulf families following the heat-induced mortality of sensitive individuals (Fig. 3C).

The functional roles of genomic associations with heat tolerance were evaluated with gene ontology (GO) enrichment analyses of genes that contained SNPs that were significantly predictive of heat survival (*n =* 2630 genes) and responded to heat selection (*n =* 257 to 387 genes) (i.e., ~a third of SNPs from the analyses above). Enrichment of biological processes and molecular functions implicated in the coral heat stress response were overrepresented in both predictive and selection gene lists (table S5), although there was limited overlap of individual GO terms among gene lists (table S5F). The enriched processes included interactions with the heat shock proteins Hsp70 and Hsp90, reflecting their cytoprotective roles and well-established up-regulation in corals under heat stress (*14*–*20*). The formation, negative regulation, and responses to reactive oxygen species (including hydrogen peroxide) and activity of the antioxidant glutathione *S*-transferase are critical processes involved in oxidative damage under heat stress in corals (*16*, *21*, *22*). These results support the heat-induced production of antioxidants in *P. daedalea* larvae and enhanced baseline antioxidant capacity in larvae from Persian Gulf versus Indian Ocean parents (*6*). The coral heat stress response also involves immunity activation and regulation (*14*, *23*), including the immunity transcription factor nuclear factor κB (NF-κB). Up-regulation of NF-κB under heat stress has been shown in several coral species (*24*–*26*), and individual heat tolerance may be predicted by baseline expression levels of NF-κB (*27*) and tumor necrosis factor (TNF) receptors involved in the activation of NF-κB (*28*, *29*). The selection responses of Persian Gulf purebreds were enriched for processes and functions linked to enhanced heat tolerance in corals (Hsp binding, glutathione *S*-transferase activity, and NF-κB interactions), which were absent in the selection responses of Indian Ocean purebreds or regional hybrids (table S5F). This finding is consistent with previously documented associations between heat tolerance and SNP variants in heat shock protein, antioxidant, and TNF receptor genes in *P. daedalea* larvae from the Persian Gulf (*30*). Additional overrepresented terms associated with coral stress responses in our *P. daedalea* gene lists include interactions with calmodulin (*17*, *30*, *31*), ubiquitin (*16*, *30*, *32*), and actin (*19*); guanosine triphosphatase activity (*26*); autophagy (*33*); and apoptosis (*34*).

Successful selective breeding for enhanced heat tolerance in the controlled setting of our experiments does not guarantee that regional hybrids would perform well in a more variable natural environment. Following our heat stress experiment, we settled out and deployed >2000 juveniles from 20 of the coral families to the Indian Ocean site at the beginning of summer (27.5°C on average). Overall survival after 28 days was characteristically low for this vulnerable life stage (*35*, *36*). However, most hybrid families (63%, *n =* 8) had at least some settled juveniles alive compared to half of the native Indian Ocean purebreds (50%, *n =* 6) and only a third of the non-native Persian Gulf purebreds (33%, *n =* 6). Total juvenile survival rates did not differ between hybrids (1.92%, *n =* 1031) and Indian Ocean purebreds (1.93%, *n =* 237) but were more than six times higher than Persian Gulf purebreds (0.31%, *n =* 1068; fig. S3). These results indicate that there may not be fitness trade-offs to selective breeding for heat tolerance, potentially due to hybrid vigor (*37*), as coral families with Persian Gulf mothers and Indian Ocean fathers exhibited strong survival performance under both laboratory heat stress and variable field conditions. However, further studies of purebred and hybrid phenotypes and their underlying genetic architecture are required to validate this interpretation. Long-term monitoring is recommended to evaluate the scope of trait enhancement and any trade-offs as corals mature [including interactive effects of microbial symbionts on coral performance (*38*–*40*)] and to assess the potential risk of outbreeding depression by tracking fitness traits across generations [i.e., when breeding occurs between evolutionarily distant populations (*41*)].

In conclusion, we show that coral genotypes from extreme environments hold the potential to enhance the thermal resilience of corals to future warming scenarios. Selective breeding with corals from the hottest reefs in the world increases thermal tolerance to temperatures not expected on most reefs until the end of the century. The strong paternal contributions to offspring heat tolerance that we observed, combined with genomic associations with heat survival, demonstrate a genetic basis for selective gains in heat tolerance. Coral gametes from warmer locations carry a larger proportion of heat-tolerant alleles owing to local adaptation (*42*, *43*), and these can enhance the tolerance of populations in cooler locations where these alleles are uncommon or rare (*44*–*46*). Introducing beneficial alleles into populations also increases the overall genetic diversity of populations (*45*) and appears to avoid phenotype-environment mismatches that occur when heat-tolerant adult corals are transplanted from warmer to cooler locations [e.g., (*47*)]. While hybridization with conspecifics from warmer locations increases offspring heat tolerance on average, our results revealed considerable variation in the contribution of individual parents to offspring performance. This includes cryptic levels of heat tolerance–associated genomic variation within a cooler population, indicating that introductions of non-native genetic material may not always be necessary to achieve selective breeding outcomes. Consequently, sexual breeding programs for trait enhancement are likely to be most successful when they incorporate a diversity of breeding combinations, particularly when individual parental genotypes and phenotypes are unknown. Introducing selectively bred corals to wild populations to assist their adaptation to climate warming involves consideration of several factors including the degree of extinction risk and standing genetic variation in the recipient population, as well as any broader ecological and socioeconomic consequences (*41*, *48*, *49*). While such intervention may benefit specific populations, immediate and ongoing actions that limit the magnitude of climate impacts and local stressors are necessary for coral reef ecosystems to persist into the future (*50*).

## MATERIALS AND METHODS

### Study sites and coral breeding

*P. daedalea* colony fragments were collected from reef sites in the southern Persian Gulf (Saadiyat Island, Abu Dhabi: 24°35′56″ N, 54°25′17″ E) and the Oman Sea in the Indian Ocean (Al Aqah Island, Fujairah: 25°29′33″ N, 56°21′49″ E), which have distinct thermal profiles (table S1). *P. daedalea* fragments were housed and spawned in controlled environment aquaria at New York University Abu Dhabi as described by Howells *et al*. (*6*). *P. daedalea* is a simultaneous hermaphrodite, and 50 full-sib families were bred from the sperm of 17 colonies (PG = 10; IO = 7) and the eggs of 4 colonies (PG = 2; IO = 2) that spawned on the third of May 2015 (fig. S1). Paternal replication was maximized in the design to focus on genetic inheritance while controlling for maternal effects. Overall, this resulted in families of Persian Gulf purebreds (18), Indian Ocean purebreds (12), and regional hybrids (20) that were crossed in a single direction to test whether Persian Gulf genes could enhance thermal tolerance. Family fertilization (0.2 to 0.3 liter, 25°C) and rearing (3 liter, after 2 to 4 hours, 27°C) was undertaken in individual sealed containers using 0.5 μm of filtered seawater at a salinity of 38.5 practical salinity units (psu) (intermediate of PG and IO values measured at the time of coral collection). Visual estimation was used to approximate an equal division of eggs from each mother (dam) and a similar density of sperm from each father (sire) for each family cross. Successful fertilization (first cleavage) was observed in all crosses after 2 hours, and no signs of fertilization were observed in eggs from each of the four colonies that did not have sperm added.

### Heat stress experiment

At 4 days of age, at the planula larval stage of development, individuals were pipetted into 300 μl of filtered seawater (0.5 μm, 38.5 psu) in matte-finish 96-well plates. For each family, 270 larvae were distributed across nine plates with the following exceptions due to low numbers of larvae: one PG family (110), one IO family (81), and two PG × IO families (30). Two families were excluded from the experiment as they did not have sufficient numbers of surviving individuals (PG84 × IO77, PG80 × IO68; table S2). Three plates per family were randomly distributed among three temperature treatments: 27°C (ambient controls), 33°C (IO-native maximum), and 36°C (PG-native maximum). The 27°C treatment was maintained in a temperature-controlled room, and the 33° and 36°C treatments were maintained in temperature-controlled incubators with initial ramping at a rate of 2°C/hour. Loggers used to monitor experimental temperatures recorded means of 26.6°, 33.2°, and 35.6°C, respectively. Survival of individuals was monitored at 0 and 60 hours using plate photographs taken in a dark room with excitation of larval fluorescent pigments to enable a relatively quick assay time for the >12,000 individuals. In later analysis of photographs, individuals were counted alive if they were fluorescently pigmented. Individuals that were nonfluorescent or not visible in two replicate photographs were counted as dead. After 60 hours, all families were returned to ambient conditions, and survivors were briefly observed for the presence or absence of motility before being fixed in RNAlater. For most family × temperature combinations, ≥10 individuals were fixed (124 of 150 cases) with the following incidence of reduced or zero samples: 27°C, 1 PG × IO; 33°C, 2 PG × IO; and 36°C, 3 PG, 9 IO, and 11 PG × IO. Tissue and/or sperm samples of all parental colonies were fixed in 100% ethanol or snap-frozen and stored at −80°C.

### Field survival experiment

An additional experiment was performed to evaluate the influence of parental origin on survivorship in the Indian Ocean field setting. In 20 of the original family cultures (6 PG, 6 IO, and 8 PG × IO), larvae were settled onto three replicate plastic tiles, threaded onto rods, and deployed 0.5 m above the substrate at 7-m depth at Dibba Island (25°36′5″ N, 56°21′9″ E, 12.5 km from the parental collection site at Al Aqah Island). Survival was monitored by microscopic counts of recruits at 0 days (end of May) and 28 days (end of June), over which the mean temperature was 27.5°C (24.4° to 30.5°C, range of daily means). Monitoring was not extended beyond this first time point because of overall low survival rates (1.4%).

### Parent and offspring genotyping

Genome-wide SNPs were targeted in adult and larval *P. daedalea* samples using 2b-RAD (*10*). DNA fragments of uniform length [36 base pairs (bp)] were generated by digesting sample extractions with *AlfI* followed by ligation with sequencing adaptors. Sample libraries were amplified in polymerase chain reaction (PCR) (17 cycles) using unique barcoded primers, and target products were extracted from agarose gels. Amplified and purified libraries were quantified with quantitative PCR and combined in equal proportions for multiplex sequencing. Pooled libraries were sequenced on an Illumina HiSeq 3000 at the Center for Genome Research and Biocomputing at Oregon State University. Demultiplexed sample reads were truncated to the restriction fragment length (36 bp), reads of poor-quality data were discarded (i.e., reads with >50% of positions with quality scores of <20), and cross_match was used to identify and remove reads matching sequencing adaptors (i.e., alignment scores of ≥18). High-quality reads were aligned to the *P. daedalea* genome (*11*) using SHRiMP and filtered to exclude weak alignments (i.e., <32 bp in length or <30 matching bp). Nucleotide frequencies at each position were called at a minimum coverage threshold of 5× for parental colonies and 50× for pooled larval samples. For parents, genotypes were scored as “distance from reference base” using a mix of coverage and minor allele frequency (MAF) thresholds. Minor alleles with <5% of reads were treated as sequencing errors and discarded. If the total remaining coverage was <5 reads, then the genotype was not called as they cannot be accurately determined. Homozygous reference was scored as 0 (96.1% of all scores), heterozygous as 0.5 (3.5%), and homozygous alternate as 1 (0.4%). For larval samples, absolute MAF values at each locus were calculated. Parent and larval datasets were further filtered to remove monomorphic loci and minimize missing data through the removal of samples and loci with poor representation. The resulting SNP datasets comprised 38,516 loci in 15 parental colonies and 38,519 loci in 87 larval samples (46 families at 27°C and 41 families at 36°C). Detailed protocols for sample preparation and scripts for genotyping are provided in (*10*, *51*, *52*).

### Data analysis

Contributions to coral heat tolerance were evaluated using end-of-experiment survival rates (i.e., 60 hours relative to 0 hours; table S2). For analyses, survival rates were analyzed as raw data, and values were standardized to ambient survival for each family to account for mortality unrelated to heat exposure. Parental origin (IO, PG, and PG × IO) effects on survival were analyzed with a linear model (relative survival ~ temperature*origin) with Tukey post hoc comparisons. In this model, data were *x*^3^-transformed to improve normality and homogeneity of variance among factor levels. Individual families that had undergone significant declines in survival at 36°C were identified using one-tailed (<1) *t* tests with adjusted *P* values (false discovery rate correction). Drivers of variation in heat tolerance were investigated in subsequent linear models including the overall contribution of both parents (i.e., family, fixed factor) and the identity of each parent (i.e., mother and father, fixed factors) on relative survival at 36°C and reported effect sizes are measures of partial eta-squared (η_p_^2^). Narrow-sense heritability (*h*^2^) of survival was estimated for each experimental temperature using Bayesian estimates of genetic variance components. Models were performed using guidelines in (*53*, *54*) on percent survival data (“Gaussian” distribution) and individual binary survival data (“threshold” distribution), with weakly informative priors (nu = 0.002, *V* = 1) and fixed residual variance (fix = 1) for the binary data. Models were run for 10^6^ iterations with the first 10^5^ discarded (burn-in) and a thinning interval of 100. Heritability was calculated as the additive genetic variance relative to the total genetic variance [i.e., *h*^2^ = *V*_A_/(*V*_A_ + *V*_R_) or *h*^2^ = *V*_A_/(*V*_A_ + *V*_M_ + *V*_R_)]. As the design of our study focused on the genetic contribution of fathers to heat tolerance, paternal effects were also calculated for each replicate while controlling for maternal influences. Specifically, heat survival (36°C, *h*) was expressed relative to each maternal mean (*m*), where survival index = (*h*/*m*) − 1. Survival data analyses were performed in R using the following packages: stats, nlme, car, nortest (models and assumptions), lsr (effect sizes), multcompView, agricolae (post hoc tests), and MCMCglmm (heritability).

Genomic variation underpinning heritable variation in thermal tolerance was evaluated with predictive associations and selection responses. To determine whether the heat survival rates of larval families could be predicted by their genetic background, we used binomial logistic regression to test whether family survival counts (alive and dead) were dependent on their baseline allele frequencies (i.e., MAF in ambient temperature samples) with parental origin (PG, IO, and PG × IO) included as a random factor. Results were then used to calculate the proportion of beneficial alleles (i.e., those that significantly predicted heat survival in larval samples) in parental colonies, where two, one, and zero copies of the allele were scored as 1, 0.5, and 0, respectively [following (*30*)]. Scores were averaged across loci and correlated with heat survival index values (above) to determine whether paternal genotype predicted offspring performance. In addition, pairwise correlations of paternal genotypes at SNPs of high effect size (*R* ≤ −0.5 and ≥ 0.5) were clustered to evaluate the genetic similarity of fathers producing tolerant versus sensitive offspring. Selection responses were assessed by binomial logistic regression of normalized sequencing reads for alleles (major and minor) in families at the end of heat exposure versus at the ambient temperature. Selection analyses were run across all families and separately for each parental origin category, excluding any families that were not represented at both temperatures. Principal components analysis (PCA) was used to visualize changes in the genetic composition of hybrid coral families relative to purebred Persian Gulf and Indian Ocean families following heat exposure. The PCA was performed on MAF values for SNPs with strong responses to heat selection (Bonferroni-corrected *P <* 0.001) and less than 10% missing data. Remaining missing values were replaced by mean population MAF values (i.e., means across IO, PG, or PG × IO samples). Genomic data analyses were performed in R using the stats, lme4, and pheatmap packages, and *P* values were adjusted for multiple testing using the Bonferroni correction. Functional enrichment analyses were performed on intragenic SNPs that were predictive of heat tolerance (adjusted *P <* 0.05) and responded to heat selection (adjusted *P <* 0.05). GO term annotations were obtained from the *P. daedalea* genome (*11*), and topGO (*55*) was used with the default settings in R. GO terms with *P* < 0.05 and occurring ≥5 times in the background set were considered significant.

## SUPPLEMENTARY MATERIALS

Supplementary material for this article is available at http://advances.sciencemag.org/cgi/content/full/7/34/eabg6070/DC1
